# Management of Internal Root Resorption on Permanent Teeth

**DOI:** 10.1155/2013/929486

**Published:** 2013-11-21

**Authors:** Elisabeth Nilsson, Eric Bonte, François Bayet, Jean-Jacques Lasfargues

**Affiliations:** ^1^Faculté de Chirurgie Dentaire, Discipline d'Odontologie Conservatrice et Endodontie, Université Paris Descartes, 1 rue Maurice Arnoux, 92120 Montrouge, France; ^2^Hôpital Bretonneau, AP-HP, 23 rue Joseph de Maistre, 75018 Paris, France

## Abstract

Internal root resorption (IRR) is a particular category of pulp disease characterized by the loss of dentine as a result of the action of clastic cells stimulated by pulpal inflammation. This review article explains the etiology, the prevalence of IRR, and, in addition to the clinical data, the contribution of the three-dimensional imaging (CBCT) to the diagnosis, the clinical decision, and the therapeutic management of IRR. The authors discussed the various therapeutic options including the orthograde or retrograde fillings of the root canal resorption area. Root canal treatment remains the treatment of choice of internal root resorption as it removes the granulation tissue and blood supply of the clastic cells. The authors describe with different clinical cases the modern endodontic techniques including optical aids, ultrasonic improvement of chemical debridement, and the use of alternative materials such as calcium silicate combined with thermoplastic filling (warm gutta-percha). In these conditions, the prognosis of the conservative treatment of internal resorptions, even if root walls are perforated, is good.

## 1. Introduction

Resorption is a condition associated with either a physiologic or a pathologic process resulting in a loss of dentin, cementum, and/or bone [1]. Root resorption may occur after various injuries, including mechanical, chemical, or thermal injury. Generally, it can be classified as internal or external root resorption. This review concerns only the internal root resorption (IRR) on permanent tooth, focusing on therapeutic options depending on the diagnosis. Internal resorption is an inflammatory process initiated within the pulp space with loss of dentin and possible invasion of the cementum [1]. Resorption phenomena have been described for many years [2]. Most of the articles on this subject focuses on external root resorptions [3], while the internal resorptions also represent a challenge for the practitioner [4]. The diagnosis of these lesions is difficult to establish and the conventional X-ray is often inadequate. Internal root radiolucencies are not detectable on radiographs at their early stages, when they are small, or because of limitations of this 2-dimensional method. Cone beam computerized tomography (CBCT) is a more powerful tool which allows an earlier and more accurate diagnosis of these lesions [5]. At the same time, new materials are offered to induce a remineralization and healing [6]. The contribution of these new ways of imaging and these new materials allow an extension of the boundaries for the conservation of teeth [7].

## 2. Pathogenesis and Histology

Internal root resorption (IRR) is a pathologic phenomenon characterized by the loss of dentine as a result of clastic cells action. It occurs in conditions of pulpal inflammation: the blood supply brings the clastic cells in the pulp chamber.

Odontoclasts (tooth resorbing cells) are morphologically analogous to osteoclasts and have similar enzymatic properties and resorption patterns. However, odontoclasts are smaller in size and form smaller resorption lacunae than osteoclasts [8]. It is unknown whether osteoclasts and tooth-resorbing cells (dentinoclasts, odontoclasts, and cementoclasts) are the same cell, but a number of similarities do exist. Odontoclasts have a ruffled border, contain fewer nuclei than osteoclasts, and have smaller or no clear zone. Both cells have intense tartrate-resistant acid phosphatase activity. The majority of odontoclasts that form lacunae on dentin are multinucleated, having 10 or fewer nuclei. Oligonuclear odontoclasts (cells with fewer than five nuclei) resorb more dentin per nucleus than do cells with a higher number of nuclei [9].

Two types of internal root resorption are generally described: the internal root canal inflammatory resorption and the internal root canal replacement resorption.

- In the inflammatory resorption, the resorptive process of the intraradicular dentin progresses without adjunctive deposition of hard tissues adjacent to the resorptive sites. The phenomenon is associated with the presence of granulation tissues in the resorbed area and identifiable with routine radiographs as a radio clear zone centered on the root canal.
- In the replacement resorption, the resorptive activity cause defects in the dentin adjacent to the root canal, with concomitant deposition of bone like tissue in some regions of the defect. It results in an irregular enlargement of the pulp space with partially or fully obliterated area of the pulp chamber.

The root resorption requires two phases: injury and stimulation. Injury is related to the nonmineralized tissues covering the internal surface of the root canal, the predentin and the odontoblasts layer. Infection is the main stimulation factor in IRR. Teeth are not symptomatic in the early stage of resorption. The origin of the resorbing cells is pulpal, coming from the apical vital part of the pulp [10]. 

## 3. Etiology

Etiology of IRR is quite unclear. Various etiologic factors have been proposed for the loss of predentin, and trauma seems to be the most advocated. In a study including 27 patients, trauma is the most common etiological factor (43%), followed by carious lesions (25%) [11]. Persistent infection of the pulp by bacteria causes the colonization of the walls of the pulp chamber by macrophage-like cells. The attachment and spreading of such cells is the primary prerequisite for initiation of root resorption [12]. It can be concluded that trauma and pulpal inflammation/infection are the major contributory factors in the initiation of internal resorption, although the complete etiologic factors as well as the pathogenesis have not yet been completely elucidated [13].

## 4. Prevalence

Internal root resorption is considered rare, but the frequency of internal resorption is not well known. Depending on the accuracy of the means evaluating the pathology, results may strongly vary. Histological studies showed a higher frequency of IRR than by a simple observation of the X-rays. The occurrence of internal resorption has been estimated to be between 0.01% and 55%, depending on the inflammatory status of the pulp [14]. A more recent histological study concluded that internal resorption was frequently detected in teeth affected by pulpitis and pulp necrosis. The lesions are not likely to be detected by conventional clinical or radiographic methods because of their small size. The development of complete pulp necrosis stops the growth of the resorption. The frequency of such lesions (concavities) offers one more reason to irrigate canals thoroughly with sodium hypochlorite during treatment [15].

## 5. Clinical and Radiographic Diagnosis (Figure 1)

Internal resorption is usually asymptomatic and often recognized clinically through routine full mouth radiographs. Pain may occur depending on the pulpal condition or perforation of the root resulting in a periodontal lesion [11]. However, clinical signs may vary according to the location of the IRR and its wideness. If the internal resorption is located in the coronal part of the canal, a clinical aspect of “pink spot” can be observed. The pink color is related to the highly vascularized connective tissue adjacent to the resorbing cells. This color turns grey/dark grey when the pulp becomes necrotic [16].

The response to vitality tests, thermal and electrical, is positive until the lesion grows significantly in size resulting in a perforation. The inflamed connective tissue filling the IRR defects degenerates, undergoes necrosis, and triggers an apical periodontitis. The tooth may then become symptomatic and periradicular abscesses may occur.

Perforation of the root is usually followed by the development of a sinus tract, which confirms the presence of an infection of the root canal, mostly by Gram-negative, strict anaerobes species [17]. 

The development of complete pulp necrosis stops the growth of the resorption because the resorbing cells are cut off from the blood supply and nutriments if the pulp chamber is sealed.

Intraoral X-ray of IRR is characterized by the radiographic appearance of an oval shape enlargement within the pulp chamber or the root canal. However the early diagnosis of the IRR is difficult by examination of a conventional X-ray. If IRR is suspected, several shots under different angles of incidence are recommended. But an accurate diagnosis is essential for an appropriate treatment plan to be devised. CBCT has been successfully used to evaluate the true nature and severity of resorption lesions in isolated case reports indicating that the clinician could more confidently diagnose and manage the defect. ROC Az values of a study comparing the accuracy of diagnosis of intraoral radiographs and the CBCT, respectively, amounted to 0.78 and 1.00, indicating the superior accuracy of CBCT [18].

The use of CBCT provides a 3-dimensional appreciation of the resorption lesion with axial, coronal, parasagittal views of the anatomy. In the serial of cross-sectional views, the size and the location of the resorption are clearly determined with high sensitivity and an excellent specificity. CBCT has a high accuracy in detecting root lesions at the earliest stages [5]. 

Sometimes, the resorption area is filled with a deposition of metaplastic hard tissue that looks like bone or cementum. This replacement resorption material has an aspect of enlargement of the pulp chamber with a fuzzy appearance of the canal space.

CBCT gives information about the following:

- location, size, and shape of the lesion,
- presence of root perforations,
- root wall thickness,
- presence of an apical bone lesion,
- localization of anatomical structures: maxillary sinus, mental foramen, and inferior alveolar nerve.

All these criteria corroborate the differential diagnosis with external root resorption and allow the prognosis assessment of the tooth, if the lesion is amendable to treatment.

## 6. Therapeutic Decision

The decision-making must take in to consideration several criteria:

- patient's age,
- tooth location,
- shape of the clinical crown,
- occlusion,
- resorption location,
- resorption wideness,
- presence or not of root perforations and their wideness,
- resistance/weakness of the remaining root hard tissue,
- periodontal status,
- ability to realize a restorative treatment on the concerned tooth.

From the information collected by clinical examination and CBCT, several options may be considered:

1. therapeutic abstention and monitoring, in absence of infectious signs and symptoms,
2. orthograde root canal treatment, with three options depending on the absence or presence of perforation of the radicular wall: complete root canal filling with gutta percha on nonperforated lesions; combined gutta percha in the root canal and MTA fillings for the perforation area; complete filling with a bioactive material (MTA or Biodentine) on apical perforated lesions located in a short root length,
3. retrograde apical treatment,
4. extraction and replacement by implants: the nonconservative treatment is indicated if the tooth is too weakened to be treated or restored.

## 7. Conservative Dental Treatments of Resorbed Teeth

Root canal treatment remains the treatment of choice of internal root resorption as it removes the granulation tissue and blood supply of the clastic cells.

Internal root resorption presents specific difficulties in instrumentation and filling.

The access cavity preparation must be as conservative as possible to preserve tooth structure and avoid further weakening of the already compromised tooth. A brisk bleeding might impair visibility in teeth with active resorbing lesions until the apical pulp tissue has been cut off and removed. The shape of the resorption defect usually makes it inaccessible to direct mechanical instrumentation [13]. 

The workinglength determination with an apex locator is not possible in case of resorptive perforation.

A great emphasis must be placed on the chemical dissolution of the vital and necrotic pulp tissue with sodium hypochlorite. The use of ultrasonic devices activates and facilitates the penetration of the irrigation solution of hypochlorite to all the areas of the root canal system [14]. The nontraumatic plastic tips of EndoActivator are particularly indicated to achieve a complete chemomechanical debridement of the root canal.

The use of calcium hydroxide as an interappointment dressing maximizes the effect of disinfection procedures, helps to control the bleeding, and necrotizes residual pulp tissue. 

About the root canal filling, the material needs to be flowable to seal the resorptive defect. Thermoplastic gutta-percha techniques seem to give the best results when the canal walls are respected.

When the root wall has been perforated, MTA is the material of choice to seal the perforation as it is biocompatible, bioactive, and well tolerated by periradicular tissues [19]. The working time can be modulated by the adjunction of water if the material starts to harden during its use.

## 8. Complete Root Canal Filling with Warm Gutta Percha (Figure 2)

This option is for IRR with no perforation of the canal walls which is the most favorable situation in long-term prognosis. The treatment is performed in two sessions.

First session is as following:

- anesthesia, rubber dam, and access cavity,
- determination of the root canal length with manual instruments,
- shaping of the canal,
- disinfection of the canal and resorption lacuna with sodium hypochlorite,
- activation of the solution with ultrasonic tips,
- drying of the canal with sterile paper tips,
- filling the canal and lacuna with calcium hydroxide as an interappointment dressing to complete the disinfection of the canal space,
- temporary sealing of the access cavity with glass ionomer cement (GIC).

Second session is as following:

- anesthesia, rubber dam, and reopening of the access cavity,
- removal of the canal calcium hydroxide by a large irrigation of ClONa activated with sonic tips,
- assessment of the root length—fitting of the guttapercha master cone,
- radiographic control to assess the good fit of the master gutta percha cone,
- final irrigation,
- drying of the root canal with sterile paper tips,
- obturation of the apical third of the root with warm gutta percha,
- gutta percha thermocompaction in the resorption lacunae to completely fill the wide canal space [20, 21],
- radiographic control,
- waterproof closing of the access cavity with a GIC.

## 9. Sealing of Internal Root Resorption with Bioactive Cements as MTA (Figures 3 and 4)

This option is indicated in presence of a perforation of the canal walls giving a communication between the root canal system and the periapical tissue. In this clinical situation, the smaller the perforation size, the more predictable the prognosis of the tooth. The treatment is performed in two sessions.

First session is as following:

- anesthesia, rubber dam placement, and access cavity,
- brisk bleeding which confirms the activity of the resorptive lesion,
- intracanal dressing with calcium hydroxide in order to dissolve necrotic soft tissue and to control the bleeding,
- sealing of the access cavity with a GIC.

Second session is as following:

- anesthesia, rubber dam placement, and access cavity,
- chemical debridement with sodium hypochlorite solution in the canal and the resorption lacuna,
- activation of the irrigant with ultrasonic tips,
- evaluation of the canal length calculated from CBCT slides and radiographic control with a gutta percha master cone,
- canal drying with upside down sterile paper tips,
- obturation of the open apex and resorption lacuna with MTA under visual control with an operative microscope,
- radiographic control of the obturation,
- placement of a water-moistened cotton pellet directly over the material,
- provisional sealing of the access cavity with a GIC.

**Figure 3:**
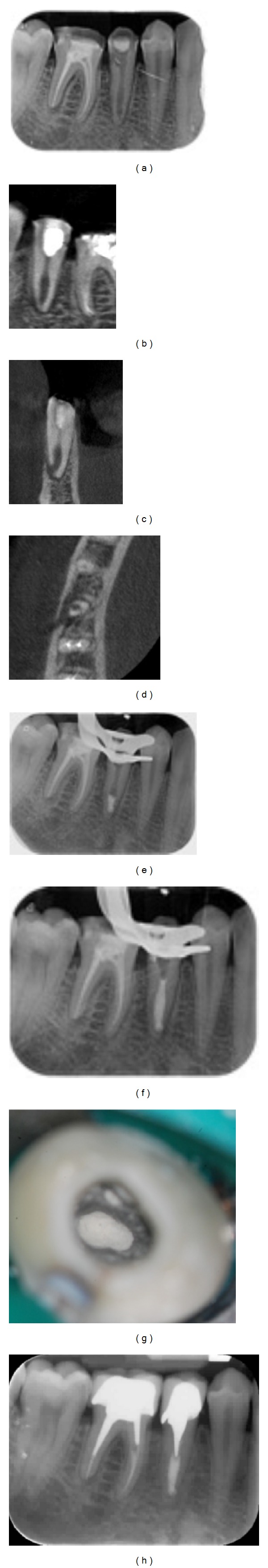
Management of internal root resorption and open apex induced using MTA cement as root canal filling material (tooth 45). (a) Preoperative intraoral radiograph of the second right lower bicuspids showing an abnormal width of the root canal in the third apical part. (b), (c), and (d) Sagittal, coronal, and axial CBCT cross-sections reveal the thickness reduction of radicular walls without perforation but with an open apex in communication with the internal resorbed part of the canal. (e), (f) To avoid an overextension of materials beyond the apex, the apical part of canal, and the resorption lacuna where both filled with MTA. (g) Clinical view with operative microscope of white MTA placed in the third apical part of the canal. (h) Periapical X-ray: control of treatment at one year. Note the absence of periapical disease and the integrity of the root.

**Figure 4:**
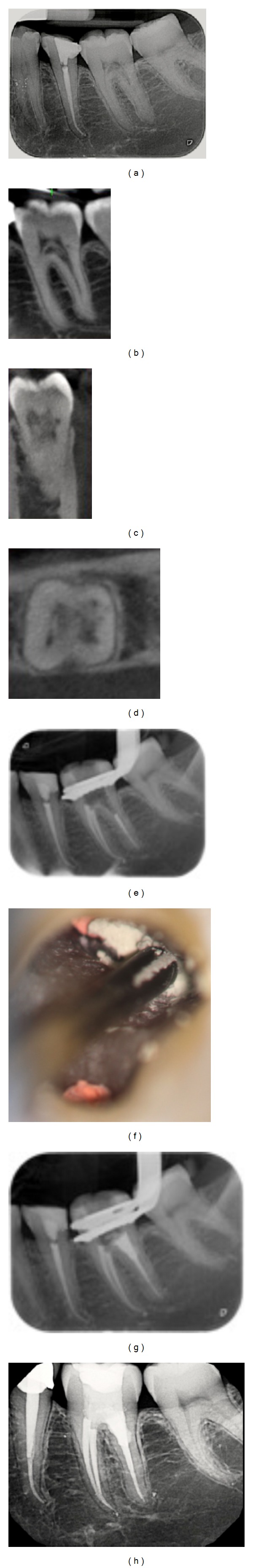
Management of internal replacement root resorption with root perforation, using both warm gutta-percha and MTA for root canal filling (tooth 36). (a) Preoperative intraoral radiograph of the first left lower molar showing an enlargement associated with a fuzzy appearance of pulp chamber and the first third of the root canal. (b), (c), and (d) Sagittal, coronal, and axial CBCT cross-sections confirm the enlargement of pulp space and the abnormal apposition of dentin/bone-like hard tissue. (e) After chemomechanical and ultrasonic removal of pathological soft and hard tissues, the mesial canals and the 2/3rd of the distal root were filled with warm compacted gutta percha, leaving free the resorbed area. (f) Peroperative clinical view of the MTA material placed in the resorbed area and (g) the immediate X-ray to assess the quality of the root canal obturation. (h) Periapical X-ray: control of treatment at one year. Note the absence of periapical disease and the healthy appearance of the furcation facing the resorbed area filled with MTA.

Considering the location of the resorption and the short length of the root, the canal can be completely filled with MTA (Figure 3). In other case, the healthy part of the canal will be filled with gutta percha (Figure 4). 

## 10. Surgical Treatment of Internal Root Resorption (Figure 5)

Surgical approach is needed when it is not possible to get access to the lesion through the canal. Surgical treatment should always be performed in a second intention, after orthograde treatment (or retreatment) has been performed, the coronal part of the canal being filled. In these cases, because of the shape of the lesion, surgical approach allows to get direct access to the lesion and to perform a mechanical cleaning of the resorbed defect. 

The general guidelines of the endodontic surgery procedure must be respected [22].

**Figure 5:**
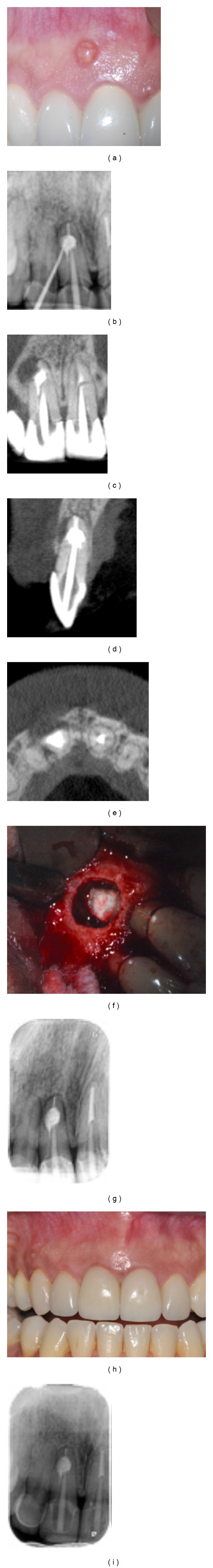
Surgical management of an internal resorption through a retrofilling with MTA (tooth 11). (a) Preoperative view: presence of a sinus tract. (b) Preoperative intraoral radiography with a gutta percha point in the sinus tract, leading to the source of infection [11]. (c), (d), and (e) Sagittal, coronal, and axial CBCT cross-sections. (f) Surgical cleaning of the root resorptive lesion and retrofilling with MTA. (g) Postoperative periapical X-ray of the root treatment: the filling is dense without overfilling. (h) One year followup: the clinical view confirms the sinus tract disappearance and the healthy appearance of the gums. (i) Periapical X-ray corroborates the periodontal regeneration in close contact with MTA filling.

Following local anesthesia a mucoperiosteal flap is raised. The cortical bone plate is removed to provide access to the root area. The softtissue lesion is curetted and the intraradicular dentin cavity is prepared with the aid of an operative microscope, cleaned, and dried. The filling materials (like MTA or Biodentine) are placed and smoothed on its external surface. The surgical procedure is finished with meticulous cleaning of the wound area. The flap is repositioned and sutured (Figure 5).

## 11. Conclusion

Internal inflammatory root resorption is a particular category of pulp disease, which can be diagnosed by clinical and radiographic examination of teeth in daily practice. Today, the diagnosis of internal root resorption is significantly improved by the three-dimensional imaging. Furthermore, the CBCT's superior diagnosis accuracy resulted in an improved management of the resorptive defects and a better outcome of conservative therapy of teeth with internal resorption. Modern endodontic techniques including optical aids, ultrasonic improvement of chemical debridement, and thermoplastic filling techniques should be used during the root canal treatment of internally resorbed teeth. Alternative materials such as calcium silicate cements offer new opportunities for the rehabilitation of resorbed teeth. In these conditions, the prognosis of the treatment of internal resorptions, even if root walls are perforated, is good.

## Figures and Tables

**Figure 1:**
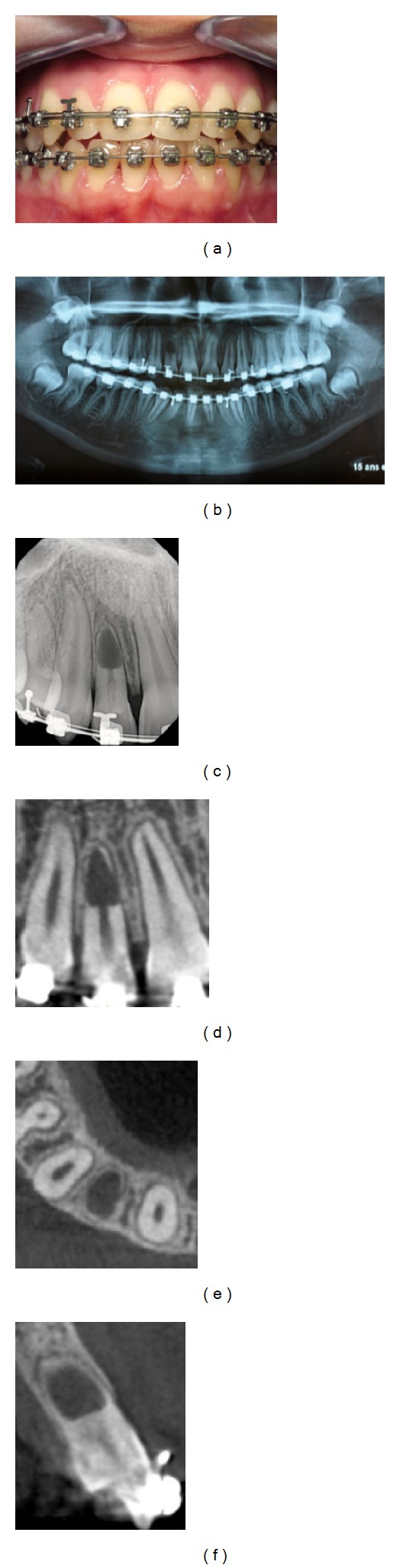
Diagnosis of an apical internal root resorption in a 16-year old patient at the end of orthodontic treatment (tooth 12). (a) Extraoral photograph: The clinical data of the examination are no pain, no crown discoloration, healthy periodontal probing, and thermic and electric pulp; vitality tests are positive (possibly false positive). (b) Panoramic radiograph: note the apical resorption of the right upper lateral incisor. (c) Periapical radiograph confirms the invasive internal resorption in the apical third: the canal disappears with perforation of radicular walls. However, the lamina dura is present. (d), (e), and (f) Sagittal, axial, and coronal CBCT cross-sections. The most likely etiological hypothesis is an inflammatory reaction of the pulp due to traumatic orthodontic procedures. Because of the absence of symptoms, the decision was the abstention with periodical clinic and radiographic controls.

**Figure 2:**
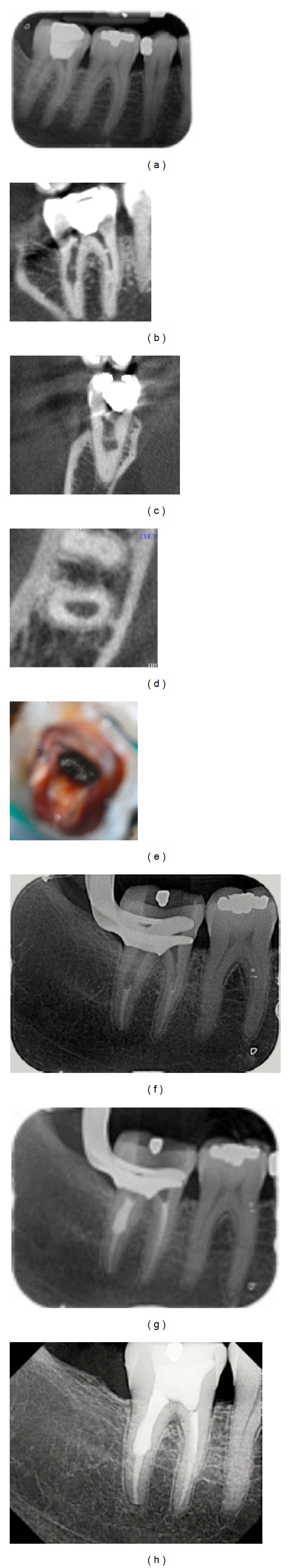
Management of internal root resorption with endodontic treatment and complete root canal filling with warm gutta percha (tooth 47). (a) Preoperative intraoral radiograph of the second right lower molar showing an abnormal width of the distal root canal. (b), (c), and (d) Sagittal, coronal, and axial CBCT cross-sections confirm the internal resorption without perforation of radicular walls. (e) Clinical aspect of the internal root defect after cleaning and shaping under operative microscope. (f) Filling of the apical third with vertical condensation of warm gutta-percha (system B Heat source, SybronEndo) and (g) warm gutta percha thermocompaction in the coronal resorbed part of the root canal. Note the density of the filling and the absence of vacuity. (h) Periapical X-ray: control of treatment at one year. Note the absence of periapical disease and the integrity of the root.
